# Factors involved in the remission of oral lichen planus treated with topical corticosteroids

**DOI:** 10.1038/s41405-024-00217-4

**Published:** 2024-05-08

**Authors:** Poosit Wongpakorn, Soranun Chantarangsu, Chanwit Prapinjumrune

**Affiliations:** 1 Department of Oral Medicine, Faculty of Dentistry, Chulalongkorn University, Bangkok, 10330 Thailand; 2 Department of Oral Pathology, Faculty of Dentistry, Chulalongkorn University, Bangkok, 10330 Thailand

**Keywords:** Oral diseases, Oral medicine

## Abstract

**Aim:**

To determine the factors that affected the complete clinical remission of oral lichen planus (OLP) treated with topical corticosteroids.

**Material and methods:**

We retrospectively evaluated the charts of patients diagnosed as OLP. Age, sex, current medical conditions, medications, type of OLP, Thongprasom score, pain level assessed by a numeric rating scale (NRS), *Candida* infection, topical steroid treatment preparation, duration of treatment until the first complete clinical remission, and follow-up duration were assessed as variables.

**Results:**

In total 100 patients, after complete remission, 22 patients reported a relapse within 1.5–45 months, with a mean of 15.6 ± 13.2 months. Age, duration, gingiva and vestibule area, hypertension, dyslipidemia, Thongprasom score, preparation and topical corticosteroid potency were factors affecting the remission. Multivariate logistic regression analysis revealed that the patients’ age and duration of treatment were significant factors after adjusted for age, sex, and independent factors with a *P*-value < 0.1 in the univariate analysis. The likelihood of having incomplete remission of the OLP lesion increased by 7.9% for every year increase in age and increased by 2.3% for every month of treatment.

**Conclusions:**

There are many different factors between the complete remission and incomplete remission groups. However, age and duration of treatment were significant factors affecting the remission of OLP.

## Introduction

Lichen planus (LP) was first described by Erasmus Wilson in 1869. The Greek word leichen, which means tree moss, and the Latin word planus, which means flat, are combined to form the term “lichen planus”. Oral lichen planus (OLP) is a chronic inflammatory disease of the oral mucosa that has been recognized as an immune pathology with an unexplained etiology [1]. However, the pathogenesis of OLP has been described to be associated with cell-mediated immune dysregulation. According to recent studies, OLP is a T-cell mediated autoimmune disease in which oral epithelial cells are killed by autocytotoxic CD8^+^ T cells with unknown specific antigens [2]. The prevalence of OLP is estimated to be ~1.01% of the global population and increases significantly and progressively after the age of 40 [3]. OLP has been classified as an oral potentially malignant disorder with a malignant transformation rate ranging from 0 to 12.5% in different studies. Although many studies support the potentially malignant character of OLP, it currently remains unresolved [4–6].

The oral manifestations of OLP typically present as a bilateral or unilateral white lace-like (reticular), atrophic or erosive lesion. Reticular lesions are often asymptomatic, while atrophic or erosive lesions are the main cause of an oral burning sensation, soreness, discomfort or pain during eating or toothbrushing. Therefore, many patients’ basic daily activities, such as eating, drinking, talking, and socializing with others are significantly restricted by OLP. Desquamative gingivitis cases have more pain and severity than other cases that can affect the patients’ esthetic appearance in cases involving the anterior gingiva [3, 7]. Lichen planus can also affect the skin, scalp, nails, genitals and other non-oral mucosa membranes [8, 9].

Although a permanent cure of OLP is not available, many treatment options have been introduced to reduce and control its painful symptoms. Topical corticosteroids are usually the first line of therapy because they have few adverse effects [10]. However, some patients who receive topical corticosteroids develop secondary candidiasis, which requires antifungal treatment [11]. Systemic corticosteroids can be used in patients with severe recalcitrant erosive OLP or patients with diffuse mucocutaneous involvement. When corticosteroids have not been effective, topical nonsteroidal immunosuppressants should be considered [12].

Because OLP is a chronic disease, complete clinical remission is difficult to achieve, including with treatment. In one study, less than 2.47% of OLP patients achieved remission, while 78% still had oral lesions at the end of the follow-up period [13]. In other studies, complete remission was reported in 28.6% of OLP patients treated with topical clobetasol with no significant adrenal suppression or adverse effects [14, 15]. 50% of patients experienced a relapse of OLP within 4–17 weeks; the mean time to relapse was 5 weeks after discontinuing treatment with topical corticosteroid ointment [16]. However, the factors associated with complete remission of OLP have not been identified. Therefore, the purpose of the present study was to determine the factors that affect the complete clinical remission of OLP treated with topical corticosteroids.

## Materials and methods

### Subjects

The study population was recruited randomly from patients who were diagnosed as OLP with modified WHO diagnostic criteria 2003 [17] from 2007 to 2023 at the Department of Oral Medicine, Faculty of Dentistry, Chulalongkorn University. The sample size calculation in this study was based on the rule of thumb [18] that the minimum ratio of observations to variables is 10:1 for a logistic regression analysis. The results indicated that 50 patients with complete healing and 50 patients with incomplete healing were required. The patients’ information was evaluated to identify subjects who were suitable for this retrospective study. The patients who were lost to follow-up for more than 6 months and discontinued using topical steroid treatment during the data collection period were excluded. This research protocol was approved by the Human Ethics Committee of the Faculty of Dentistry, Chulalongkorn University, Bangkok, Thailand (HREC-DCU 2022-100). Informed consent from participants was waived by the Human Ethics Committee that approved this study.

### Variables

As shown in Table 1, age, sex, current medical conditions, medications, type of OLP (reticular, plaque-like, atrophic, erosive/ulcerative, papular and bullous), Thongprasom score [19], pain level assessed by a numeric rating scale (NRS) [20], presence of *Candida* infection, topical steroid treatment preparation (oral paste, gel, mouthwash), duration of treatment until the first complete clinical remission, and follow-up duration (from index date to final visit within the study period) were recorded for each patient in the study. Complete clinical remission was defined as the absence of symptoms (numerical rating scale = 0/10) and the remission of all atrophic/erosive lesions regardless of any white lesions (Thongprasom score = 0). Partial remission was defined as a decrease in, but not the complete remission, of atrophic/erosive areas and symptoms.

**Table 1:** Variables.

No.	Variables
1	Age
2	Sex
3	Medical conditions
4	Medications
5	Type of OLP
6	Thongprasom score
7	NRS
8	*Candida* infection
9	Topical steroid treatment
10	Follow-up duration

### Statistical analysis

The statistical analyses were performed using IBM SPSS Statistics version 29.0 (IBM corporation, Armonk, NY, USA) at a significance level of 5%. For numerical factors, descriptive statistics are reported as means and standard deviations (SD) or medians and interquartile range (IQR), as appropriate. For categorical factors, frequencies and percentages are described. Differences between the complete and incomplete groups were analyzed by the Chi-square test or Fisher’s exact test for categorical factors and independent *t*-test or Mann–Whitney *U* test for numerical factors. The univariate and multivariate logistic regression analyses were used to investigate the factors affecting incomplete healing of OLP. The final multivariate logistic regression model was constructed by including age, sex, and independent factors with a *P*-value < 0.1 in the univariate analysis. The results of the logistic regression analyses were expressed as the odds ratio (OR) with a 95% confidence interval (95% CI).

## Results

### Patient demographic data

One hundred patients were randomized for this study. There were 50 patients with complete remission and 50 patients with incomplete remission. Among the OLP patients, 88 were female (88.0%) and 12 were male (12.0%), resulting in a female-to-male ratio greater than 7:1. The patients’ age ranged from 18 to 74 years old, with a mean age of 52.7 ± 11.1 years. Sex was not significantly different between subgroups (*P* = 0.065). In contrast, age was significantly different. The mean age of the patients with incomplete remission group was 56.2 ± 10.0, in contrast that of the patients in the complete remission group was 49.3 ± 11.1, which was significantly different (*P* = 0.002). The follow-up duration ranged from 1.5 to 167.5 months with a mean of 47.7 ± 43.6 months. The follow-up duration was significantly longer in the patients with incomplete remission. After complete remission, 22 (44.0%) patients reported an OLP relapse within 1.5–45 months with a mean of 15.6 ± 13.2 months (Table 2).

**Table 2:** Demographic data of the patients.

Characteristics	Complete remission (*N* = 50)	Incomplete remission (*N* = 50)	Total (*N* = 100)	*P*-value
Sex, *n* (%)				0.065^a^
Male	9 (18.0)	3 (6.0)	12 (12.0)	
Female	41 (82.0)	47 (94.0)	88 (88.0)	
Age (year), mean ± SD	49.3 ± 11.1	56.2 ± 10.0	52.7 ± 11.1	**0.002**^**b**^
Duration (month), median (IQR)	22.3 (46.9)	49.5 (72.3)	30.3 (60.4)	**0.016**^**c**^

Statistically significant association with demographic data of the patients according to the remission of the oral lichen planus lesion (*P*-value < 0.05) is indicated in bold.

^a^Chi-square test.

^b^Independent *t*-test.

^c^Mann–Whitney *U* test.

### General characteristics of the lesion

Most of the OLP lesions were observed in the buccal mucosa (84.0%), follow by the gingiva (58.0%), vestibule (56.0%), tongue (27.0%), lips (16.0%), palate (8.0%) and labial mucosa (2.0%). OLP in the gingiva and vestibule was significantly higher in the incomplete group compared with the complete remission group (*P* = 0.005 and *P* = 0.044, respectively). Although the type of OLP did not show a significant difference between groups (*P* = 0.117), more than 90% of the lesions were mixed red and white lesions, with 34% being erosive lesions. Furthermore, erosive lesions were found more frequently in patients with incomplete remission than in the complete remission group (34% vs. 18%). The medical conditions were also significantly different between groups. There were significantly more patients with hypertension and dyslipidemia (DLP) in the incomplete remission group compared with the complete remission group (*P* = 0.032 and *P* = 0.006, respectively). The Thongprasom scores were significantly different between groups (*P* = 0.013). The Thongprasom score in the complete remission group ranged from 1 to 3, while the range in the incomplete remission group was 3–5. In contrast, the pain NRS was not significantly different between the complete and incomplete remission groups (*P* = 0.941) (Table 3).

**Table 3:** General characteristics of the lesion.

Characteristics	Complete remission (*N* = 50)	Incomplete remission (*N* = 50)	Total (*N* = 100)	*P*-value
Type of OLP, *n* (%)				0.117^a^
White	4 (8.0)	0 (0.0)	4 (4.0)	
Mixed	46 (92.0)	50 (100.0)	96 (96.0)	
Location, *n* (%)				
Buccal mucosa	44 (88.0)	40 (80.0)	84 (84.0)	0.275^b^
Gingiva	22 (44.0)	36 (72.0)	58 (58.0)	**0.005**^**b**^
Tongue	14 (28.0)	13 (26.0)	27 (27.0)	0.822^b^
Vestibule	23 (46.0)	33 (66.0)	56 (56.0)	**0.044**^**b**^
Palate	2 (4.0)	6 (12.0)	8 (8.0)	0.269^a^
Labial mucosa	1 (2.0)	1 (2.0)	2 (2.0)	1.000^a^
Lips	6 (12.0)	10 (20.0)	16 (16.0)	0.275^a^
Medical history, *n* (%)				
Hypertension	11 (22.0)	21 (42.0)	32 (32.0)	**0.032**^**b**^
Diabetes mellitus	2 (4.0)	7 (14.0)	9 (9.0)	0.160^a^
Thyroid disease	1 (2.0)	5 (10.0)	6 (6.0)	0.204^a^
DLP	11 (22.0)	24 (48.0)	35 (35.0)	**0.006**^**b**^
Cardiovascular disease	3 (6.0)	6 (12.0)	9 (9.0)	0.487^a^
GI disease	5 (10.0)	4 (8.0)	9 (9.0)	1.000^a^
Liver disease	2 (4.0)	2 (4.0)	4 (4.0)	1.000^a^
Malignancy	2 (4.0)	0 (0.0)	2 (2.0)	0.495^a^
Others	4 (8.0)	9 (18.0)	13 (13.0)	0.137^b^
NRS, median (IQR)	5.0 (3.0)	5.0 (4.0)	5.0 (4.0)	0.941^c^
Thongprasom score				
median (IQR)	3.0 (1.0)	3.0 (1.0)	3.0 (1.0)	**0.013**^**c**^
Score, *n* (%)				
Score 1	4 (8.0)	0 (0.0)	4 (4.0)	
Score 2	10 (20.0)	6 (12.0)	16 (16.0)	
Score 3	27 (54.0)	27 (54.0)	54 (54.0)	
Score 4	8 (16.0)	13 (26.0)	21 (21.0)	
Score 5	1 (2.0)	4 (4.0)	5 (5.0)	
*Candida* (time), median (IQR)	0.0 (1.0)	1.0 (2.0)	0.0 (2.0)	0.118^c^

Statistically significant association with general characteristics of the patients according to the remission of the oral lichen planus lesion (*P*-value < 0.05) is indicated in bold.

^a^Fisher’exact test.

^b^Chi-square test.

^c^Mann–Whitney U test.

### Topical steroid treatment

Variables related to the topical steroid treatments are shown in Table 4. Most of the patients with complete remission used an oral paste, while the patients with incomplete remission used a mouthwash. There were 8 patients (16.0%) in the incomplete remission group who used 0.1% triamcinolone acetonide mouthwash, which was higher than the patients in the complete remission group (*P* = 0.006). In contrast, 27 patients (54.0%) with complete remission used 0.1% Fluocinolone acetonide oral paste (FAO), which was higher than the patients in the incomplete remission group (*P* = 0.026).

**Table 4:** Topical steroid treatment.

Type of topical steroid, *n* (%)	Complete remission (*N* = 50)	Incomplete remission (*N* = 50)	Total (*N* = 100)	*P*-value
0.05% Dexamethasone mouthwash	7 (14.0)	13 (26.0)	20 (20.0)	0.134^a^
0.1% Triamcinolone mouthwash	0 (0.0)	8 (16.0)	8 (8.0)	**0.006**^**b**^
0.1% Triamcinolone acetonide oral paste	3 (6.0)	1 (2.0)	4 (4.0)	0.617^b^
0.05% Fluocinolone acetonide mouthwash	1 (2.0)	7 (14.0)	8 (8.0)	0.059^b^
0.1% Fluocinolone acetonide solution (FAS)	2 (4.0)	3 (6.0)	5 (5.0)	1.000^b^
0.1% Fluocinolone acetonide oral paste (FAO)	27 (54.0)	16 (32.0)	43 (43.0)	**0.026**^**a**^
0.05% Clobetasol propionate oral paste	2 (4.0)	0 (0.0)	2 (2.0)	0.495^b^
0.1% Fluocinolone acetonide + 1% Clotrimazole oral gel	12 (24.0)	10 (20.0)	22 (22.0)	0.629^a^

Statistically significant association with topical steroid treatment of the patients according to the remission of the oral lichen planus lesion (*P*-value < 0.05) is indicated in bold.

^a^Chi-square test.

^b^Fisher’s exact test.

### Univariate and multivariate logistic regression

The results of the univariate and multivariate logistic regression analyses performed to evaluate the factors influencing the incomplete remission of the OLP lesion are shown in Table 5. The final multivariate logistic regression analysis revealed that the patients’ age and treatment duration were significant factors influencing the incomplete remission of the OLP lesion after adjusting for age, sex, and independent factors with *P*-value < 0.1 in the univariate analysis. The likelihood of having incomplete remission of the OLP lesion increased by 7.9% for every year increase in age (OR: 1.079; 95% CI: 1.020–1.141; *P* = 0.008) and increased by 2.3% for every month of treatment (OR: 1.023; 95% CI: 1.008–1.039; *P* = 0.002).

**Table 5:** Univariate and multivariate logistic regression of factors affecting incomplete healing of oral lichen planus.

Factors	Univariate analysis		Multivariate analysis	
	OR (95% CI)	*P*-value	OR (95% CI)	*P*-value
Age (year)	1.064 (1.022–1.109)	**0.003**^a^	1.079 (1.020–1.141)	**0.008**
Female	3.439 (0.872–13.563)	0.078^a^	2.722 (0.464–15.955)	0.267
NRS scoring at 1st visit	1.013 (0.882–1.163)	0.860		
Thongprasom scoring at 1st visit	2.001 (1.183–3.386)	**0.010**^a^	1.651 (0.813–3.351)	0.165
*Candida* infection (time)	1.186 (0.902–1.559)	0.223		
Duration of treatment (month)	1.014 (1.003–1.024)	**0.009**^a^	1.023 (1.008–1.039)	**0.002**
Type of oral lichen planus				
White	Reference|			
Mixed	N/A	–		
Location of oral lichen planus				
Buccal mucosa	0.545 (0.182–1.637)	0.280		
Gingiva	3.273 (1.424–7.524)	**0.005**^a^	2.182 (0.723–6.583)	0.166
Tongue	0.903 (0.373–2.186)	0.822		
Vestibule	2.279 (1.017–5.108)	**0.046**^a^	2.899 (0.930–9.034)	0.067
Palate	3.273 (0.627–17.071)	0.159		
Labial mucosa	1.000 (0.061–16.444)	1.000		
Lips	1.833 (0.611–5.502)	0.280		
Medical history				
Hypertension	2.567 (1.072–6.150)	**0.034**^a^	1.098 (0.300–4.022)	0.888
Diabetes mellitus	3.907 (0.770–19.831)	0.100		
Thyroid	5.444 (0.612–48.397)	0.128		
DLP	3.273 (1.372–7.806)	**0.008**^a^	1.622 (0.442–5.950)	0.466
Cardiovascular disease	2.136 (0.503–9.068)	0.303		
GI disease	0.783 (0.197–3.103)	0.727		
Liver disease	1.000 (0.135–7.392)	1.000		
Malignancy	N/A	–		
Others	2.524 (0.723–8.818)	0.147		
Type of topical steroid				
0.05% Dexamethasone mouthwash	2.158 (0.779–5.977)	0.139		
0.1% Triamcinolone acetonide mouthwash	N/A	–		
0.1% Triamcinolone acetonide oral paste	0.320 (0.032–3.184)	0.331		
0.05% Fluocinolone acetonide mouthwash	7.977 (0.943–67.456)	0.057^a^	7.186 (0.608–84.927)	0.118
0.1% Fluocinolone acetonide solution (FAS)	1.532 (0.245–9.587)	0.648		
0.1% Fluocinolone acetonide oral paste (FAO)	0.401 (0.178–0.905)	**0.028**^a^	0.424 (0.148–1.215)	0.110
0.05% Clobetasol propionate oral paste	N/A	–		
0.1% Fluocinolone acetonide +1% Clotrimazole oral gel	0.792 (0.306–2.046)	0.630		

Statistically significant association with affecting incomplete remission of OLP (*P*-value < 0.05) is indicated in bold.

*OR* odds ratio, *CI* confidence interval, *N/A* not applicable.

^a^Independent factors with *P*-value < 0.1 in the univariate analysis and was included in the final multivariate logistic regression model.

## Discussion

In our study, we determined the factors that affected the complete clinical remission of OLP lesions treated with topical corticosteroids. The OLP lesions in this study were mostly found in females, resulting in a female-to-male ratio greater than 7:1. The age of this study group ranged from 18 to 74 years old with a mean age 52.7 ± 11.1 years. In other demographic studies, the highest prevalence for males was found in 65–74 year-olds and 55–64 year-old females. In contrast, the prevalence was low among the younger age groups. The prevalence among females was significantly higher than among males, predominantly affecting those ≥40 years old. [21, 22]. Our findings concluded that OLP remission was not significantly different between sexes. In contrast, other studies found that the female sex was one of the predictors of symptomatic OLP; females had a higher pain level compared with males [23, 24]. Furthermore, females had more symptoms than males [13].

OLP remission was also significantly influenced by age. Our results revealed that the younger age group had in a higher prevalence of complete remission compared with the older age group. There was also a 7.9% increased risk of incomplete healing with every additional year. This was due to elderly patients having more medical conditions and lower treatment compliance than younger patients. Many drugs also have adverse effects on the oral mucosa, weakening the tissues and resulting in a worse therapeutic outcome. Moreover, the oral mucosa changes and lower treatment cooperation in the elder may negatively impact the successful management of OLP. Finally, metabolic changes due to aging, as well as weakened immunity, nutritional deficits, drug use, or denture wearing may impact treatment success [22, 25]. Another study found that OLP lesions were most prevalent in patients in the 50–60-year age group. However, more new lesion formations were observed in patients younger than 50 years old [7].

More than 90% of OLP patients in this study exhibited a mixed red and white lesion. The type of OLP lesions determined in this study is similar to a previous study [24]. Erosive lesions were frequently observed in the incomplete remission group. Previous studies found that erythematous/erosive and ulcerative lesions tend to become more painful and symptomatic compared with the white/plaque-type lesion [24, 26]. In the present study, the type of OLP did not result in different treatment outcomes between groups. However, clinically categorizing the severity of OLP using the Thongprasom scoring system demonstrated a significant association between groups. At the first visit, the complete remission group had a score of 1–3, which means a white to erythematous lesion, while the incomplete remission patients had a score of 3–5, which means an erythematous to erosive lesion. The results from the present study indicated that the more severe the lesion on the first visit, the more likely there is an incomplete remission, similar to a previous study that found the reticular type had a better prognosis than the erosive type which does not heal spontaneously [27].

Most OLP lesions in this study were found in the buccal mucosa, similar to other studies [8, 23, 26, 28, 29]. Gingival and vestibule involvement were the second and the third most common site. Interestingly, the results from this study demonstrated unfavorable treatment outcomes of OLP located in the gingival and vestibular areas were similar to those of a previous study [7]. When OLP affects the gingiva by presenting as an atrophic or ulcerative form, toothbrushing becomes difficult due to pain and bleeding. The patients then frequently present with an accumulation of dental plaque and calculus, which negatively affects OLP remission. Poor oral hygiene results in increased bacteria deposition, which promotes an inflammatory response that aggravates the OLP lesions. The amounts of periodontopathogens were observed to be greater in OLP patients than in non-OLP patients [30]. Two hypotheses can explain the relationship between OLP and periodontopathogenic microorganisms. The first is an increase in periodontopathogenic microorganisms following the formation of OLP lesions. Dental plaque and calculus are local irritant factors that prevent OLP lesion healing and change the characteristics of the lesions to be more aggressive. The second is that periodontopathogenic microorganisms play a direct role in the etiology of OLP and induce the formation of OLP lesions [30]. However, the topical steroid was difficult to apply and had less contact in the vestibular area. These reasons might explain why the gingival and vestibule area have a poor prognosis. A mouthwash preparation is recommended rather than an ointment considering the difficulty of placing the ointment or gel, especially in the vestibule area [7, 31].

Regarding medical conditions and medications, our study found that patients with hypertension, diabetes mellitus (DM), and DLP were highly frequent observed in the incomplete remission group. Hypertension was the most common disease, followed by diabetes mellitus. Currently, many medications have been reported to cause various kinds of oral lesions. Antihypertensive drugs were the most commonly used drugs in Thai OLP patients, followed by hypoglycemic drugs that could result in oral lichenoid drug reactions (OLDR) [32, 33]. Moreover, OLP may be a manifestation of systemic disease. In early 1963, Grinspan reported Grinspan’s syndrome, which is a triad of conditions, i.e., essential vascular hypertension, diabetes mellitus, and lichen planus of the oral mucosa [34]. Statins are hypolipidemic drugs frequently used in to treat DLP patients. The adverse effects of simvastatin were first described by Roger et al. in 1994 when it caused lichenoid drug reactions on the skin and oral mucosa [35]. Another study showed that simvastatin may be associated with severe oral lesions when combined with amlodipine [36]. Currently, a wide range of drugs are known to cause oral lichenoid lesion (OLL), however confirming the diagnosis of OLDR remains difficult [31, 33, 37]. Although hypertension and DLP were significantly different between the complete and incomplete remission groups in this study, they had no effect on OLP remission.

Currently, topical corticosteroids are widely used for treating OLP patients. For use in the oral cavity, they are available in adhesive forms or as a solution. Empirical evidence suggests that mouthwashes are beneficial in patients with widespread symptomatic OLP where the lesions are unreachable for applying ointments or gels [9]. Interestingly, the present study found a significant difference in the preparation and potency of topical steroids between groups. 0.1% Triamcinolone acetonide (0.1%TA) mouthwash was used frequently in the incomplete remission group, whereas OLP patients with complete remission predominantly used 0.1% FAO. Deciding on the potency of the selected topical corticosteroid is crucially important when treating OLP patients. A previous study also demonstrated high success when treating OLP patients with topical fluocinolone acetonide compared with topical triamcinolone acetonide [19]. Furthermore, another study reported that after two years of treatment, 77.3, 21.4, and 17.0% of patients treated with FAO, Fluocinolone acetonide solution (FAS), and FAS/FAO, respectively, experienced complete remission [38]. Another commonly used topical corticosteroid for treating OLP is topical clobetasol, a high potency corticosteroid, that has not demonstrated significant adrenal suppression or side effects [14]. Although our results showed no significant difference in successfully treating OLP with topical clobetasol between groups, complete remission in 28.6% of OLP patients treated with clobetasol was reported [15]. Consequently, topical corticosteroids may be a useful, safe, and effective alternative therapy for OLP. In contrast, patients treated with topical corticosteroids might be at risk of develop oral candidiasis, which was the most frequent side effect and requires antifungal treatment [11, 31]. However, systemic corticosteroids may be used in OLP patients with severe erosive lesions or diffuse mucocutaneous involvement and, when corticosteroids fail, topical nonsteroidal immunosuppressants should be used [12].

While treating oral candidiasis, steroid treatment should be stopped, which results in the worsening of the lesions. An orabase preparation of a moderate potency corticosteroid is most commonly used as a standard topical treatment in the oral cavity can promote *Candida* growth and the recurrence of oral candidiasis. The increased incidence of *Candida* infection in the oral cavity of OLP patients undergoing topical corticosteroid treatment has been reported in several studies [38–41]. However, the amount of *Candida* from the oral cavity of the OLP patients before and after treatment with 0.1% FAO was not significantly different [42]. Similar to a previous study, our results showed that oral *Candida* infection did not affect OLP remission. Furthermore, another study reported no significant difference between the clinical features of OLP and *Candida* infection [43].

Typically, the clinical features of OLP can undergo relapse and remission. During an exacerbation, the OLP symptoms and clinical signs will increase; in contrast, during a remission, the symptoms and signs will decrease [2]. The mean duration of oral lichen planus in an exacerbation period is 60 months [27]. Even with treatment, complete remission of OLP is difficult to achieve. It has been reported that 50% of OLP patients had a relapse of OLP within 4–17 weeks with a mean time of 5 weeks after stopping topical steroid treatment [16]. Another study reported that less than 2.47% of OLP patients achieved remission, while 78% still had oral lesions at the end of the follow-up period [13]. Importantly, the treatment duration had a significant impact on the remission of OLP. The longer the patient was treated, the more they had a prognosis of incomplete remission. Although the pathogenesis of OLP remains unclarified, destruction of the basal keratinocytes disrupts the basement membrane homeostasis, blocking normal cell survival signaling and leads to apoptosis and cell death resulting in the chronicity of OLP [44]. In the present study, the patients reported a relapse of OLP within 1.5–45 months with a mean of 15.6 ± 13.2 months after complete remission. The duration of disease in the relapse and remission periods varied, which might depend on the extent and site of involvement and morphology of the lesions. The management of OLP is still not totally satisfactory in all cases and there is as yet no definitive treatment that results in permanent remission.

## Conclusions

In conclusion, the present study demonstrated that age, duration, location in the gingiva and vestibule, hypertension, dyslipidemia, Thongprasom score, 0.1% triamcinolone acetonide mouthwash, and FAO were factors affecting OLP remission. However, age and treatment duration were significant factors that affected the remission of OLP. Additionally, periodontal treatment in conjunction with topical steroids was beneficial for promoting the successful treatment of gingival OLP lesions.

## Data Availability

The data supporting the findings of this study are available from the corresponding author upon request.

## References

[1] Warnakulasuriya S, Johnson NW, Van der Waal I (2007). Nomenclature and classification of potentially malignant disorders of the oral mucosa. J Oral Pathol Med.

[2] Ismail SB, Kumar SK, Zain RB (2007). Oral lichen planus and lichenoid reactions: etiopathogenesis, diagnosis, management and malignant transformation. J Oral Sci.

[3] González-Moles MÁ, Warnakulasuriya S, González-Ruiz I, González-Ruiz L, Ayén Á, Lenouvel D (2021). Worldwide prevalence of oral lichen planus: A systematic review and meta‐analysis. Oral Dis.

[4] Warnakulasuriya S, Kujan O, Aguirre-Urizar JM, Bagan JV, González-Moles MÁ, Kerr AR (2021). Oral potentially malignant disorders: A consensus report from an international seminar on nomenclature and classification, convened by the WHO Collaborating Centre for Oral Cancer. Oral Dis.

[5] Kurago ZB (2016). Etiology and pathogenesis of oral lichen planus: an overview. Oral Surg Oral Med Oral Pathol Oral Radiol.

[6] Gonzalez‐Moles M, Scully C, Gil‐Montoya J (2008). Oral lichen planus: controversies surrounding malignant transformation. Oral Dis.

[7] Park S-Y, Lee H-J, Kim S-H, Kim SB, Choi YH, Kim YK (2018). Factors affecting treatment outcomes in patients with oral lichen planus lesions: a retrospective study of 113 cases. J Periodontal Implant Sci.

[8] Gümrü B (2013). A retrospective study of 370 patients with oral lichen planus in Turkey. Medicina oral, patología oral y cirugía bucal. Med Oral Patol Oral Cir Bucal.

[9] Alrashdan MS, Cirillo N, McCullough M (2016). Oral lichen planus: a literature review and update. Arch Dermatol Res.

[10] Carrozzo M, Porter S, Mercadante V, Fedele S (2019). Oral lichen planus: A disease or a spectrum of tissue reactions? Types, causes, diagnostic algorhythms, prognosis, management strategies. Periodontology.

[11] Eisen D, Carrozzo M, Bagan Sebastian JV, Thongprasom K (2005). Number V Oral lichen planus: clinical features and management. Oral Dis.

[12] Scully C, Eisen D, Carrozzo M (2000). Management of oral lichen planus. Am J Clin Dermatol.

[13] Carbone M, Arduino PG, Carrozzo M, Gandolfo S, Argiolas MR, Bertolusso G (2009). Course of oral lichen planus: a retrospective study of 808 northern Italian patients. Oral Dis.

[14] Carbone M, Conrotto D, Carrozzo M, Broccoletti R, Gandolfo S, Scully C (1999). Topical corticosteroids in association with miconazole and chlorhexidine in the long‐term management of atrophic‐erosive oral lichen planus: a placebo‐controlled and comparative study between clobetasol and fluocinonide. Oral Dis.

[15] Dillenburg CS, Martins MA, Munerato MC, Marques MM, Carrard VC, Sant’Ana Filho M (2014). Efficacy of laser phototherapy in comparison to topical clobetasol for the treatment of oral lichen planus: a randomized controlled trial. J Biomed Opt.

[16] Kazancioglu HO, Erisen M (2015). Comparison of low-level laser therapy versus ozone therapy in the treatment of oral lichen planus. Ann Dermatol.

[17] Van der Meij E, Van der Waal I (2003). Lack of clinicopathologic correlation in the diagnosis of oral lichen planus based on the presently available diagnostic criteria and suggestions for modifications. J Oral Pathol Med.

[18] Hair JF, Black WC, Babin BJ, Anderson RE, Tatham RL (2006). Multivariate data analysis 6th Edition. Pearson Prentice Hall. New Jersey. humans: Critique and reformulation. J Abnorm Psychol.

[19] Thongprasom K, Luangjarmekorn L, Sererat T, Taweesap W (1992). Relative efficacy of fluocinolone acetonide compared with triamcinolone acetonide in treatment of oral lichen planus. J Oral Pathol Med.

[20] Downie WW, Leatham PA, Rhind VM, Wright V, Branco JA, Anderson JA (1978). Studies with pain rating scales. Ann Rheum Dis.

[21] Axéll T, Rundquist L (1987). Oral lichen planus–a demographic study. Community Dent Oral Epidemiol.

[22] Li C, Tang X, Zheng X, Ge S, Wen H, Lin X (2020). Global prevalence and incidence estimates of oral lichen planus: a systematic review and meta-analysis. JAMA Dermatol.

[23] Budimir V, Richter I, Andabak-Rogulj A, Vučićević-Boras V, Budimir J, Brailo V (2014). Oral lichen planus–Retrospective study of 563 Croatian patients. Med Oral Patol Oral Cir Bucal.

[24] Osipoff A, Carpenter MD, Noll JL, Valdez JA, Gormsen M, Brennan MT (2020). Predictors of symptomatic oral lichen planus. Oral Surg Oral Med Oral Pathol Oral Radiol.

[25] Gönül M, Gül U, Kaya İ, Koçak O, Cakmak SK, Kılıç A (2011). Smoking, alcohol consumption and denture use in patients with oral mucosal lesions. J Dermatol Case Rep.

[26] Radochová V, Dřízhal I, Slezák R (2014). A retrospective study of 171 patients with oral lichen planus in the East Bohemia-Czech Republic–single center experience. J Clin Exp Dent.

[27] Daoud MS, Pittelkow MR. Lichen Planus. In: Goldsmith LA, Katz SI, Gilchrest BA, Paller AS, Leffell DJ, Wolff K, editors. Fitzpatrick’s Dermatology in General Medicine. 8th ed, New York: The McGraw-Hill Companies; 2012.

[28] Lauritano D, Arrica M, Lucchese A, Valente M, Pannone G, Lajolo C (2016). Oral lichen planus clinical characteristics in Italian patients: a retrospective analysis. Head Face Med.

[29] Ingafou M, Leao JC, Porter SR, Scully C (2006). Oral lichen planus: a retrospective study of 690 British patients. Oral Dis.

[30] Seckin Ertugrul A, Arslan U, Dursun R, Sezgin Hakki S (2013). Periodontopathogen profile of healthy and oral lichen planus patients with gingivitis or periodontitis. Int J Oral Sci.

[31] Al-Hashimi I, Schifter M, Lockhart PB, Wray D, Brennan M, Migliorati CA (2007). Oral lichen planus and oral lichenoid lesions: diagnostic and therapeutic considerations. Oral Surg Oral Med Oral Pathol Oral Radiol Endodontol.

[32] Thongprasom K, Youngnak-Piboonratanakit P, Pongsiriwet S, Laothumthut T, Kanjanabud P, Rutchakitprakarn L (2010). A multicenter study of oral lichen planus in Thai patients. J Investig Clin Dent.

[33] Lamey PJ, Gibson J, Barclay SC, Miller S (1990). Grinspan’s syndrome: a drug-induced phenomenon?. Oral Surg Oral Med Oral Pathol.

[34] Grinspan D, Diaz J, Villapol LO, Schneiderman J, Berdichesky R, Palèse D (1966). Lichen ruber planus of the buccal mucosa. Its association with diabetes. Bull Soc Fr Dermatol Syphiligr.

[35] Roger D, Rolle F, Labrousse F, Brosset A, Bonnetblanc JM (1994). Simvastatin‐induced lichenoid drug eruption. Clin Exp Dermatol.

[36] Thongprasom K (2018). Is Simvastatin Associated with Oral Lichenoid Drug Reaction?. J Dent Indones.

[37] McCartan B, McCreary C (1997). Oral lichenoid drug eruptions. Oral Dis.

[38] Thongprasom K, Luengvisut P, Wongwatanakij A, Boonjatturus C (2003). Clinical evaluation in treatment of oral lichen planus with topical fluocinolone acetonide: a 2‐year follow‐up. J Oral Pathol Med.

[39] Jainkittivong A, Kuvatanasuchati J, Pipattanagovit P, Sinheng W (2007). Candida in oral lichen planus patients undergoing topical steroid therapy. Oral Surg Oral Med Oral Pathol Oral Radiol Endodontol.

[40] Lodi G, Tarozzi M, Sardella A, Demarosi F, Canegallo L, Di Benedetto D (2007). Miconazole as adjuvant therapy for oral lichen planus: a double‐blind randomized controlled trial. Br J Dermatol.

[41] Marable DR, Bowers LM, Stout TL, Stewart CM, Berg KM, Sankar V (2016). Oral candidiasis following steroid therapy for oral lichen planus. Oral Dis.

[42] Saengprasittichok N, Sucharitakul J, Matangkasombut O, Prapinjumrune C (2022). Effect of fluocinolone acetonide (0.1%) treatment in oral lichen planus patients on salivary lactoferrin levels and Candida colonization: a prospective study. BMC Oral Health.

[43] Parlatescu I, Nicolae C, Tovaru S, Radu L, Penes O, Varlas V (2021). The Implication of Candida Infection in Oral Lichen Planus Lesions. Maedica.

[44] Mangold AR, Pittelkow MR. Lichen planus. In: Gaspari AA, Kaplan DH, Tyring SK, editors. Clinical and Basic Immunodermatology. 2nd ed. pp 551–76. Cham: Springer International Publishing; 2017.

